# Announcement

**DOI:** 10.1111/acel.12292

**Published:** 2014-11-24

The NIA Interventions Testing Program Announces the 2015 Solicitation of Proposals

The National Institute on Aging (NIA) Interventions Testing Program (ITP) investigates dietary supplements purported to extend lifespan and/or delay the onset of disease and disability. The NIA ITP tests such compounds in mice, using a variety of measured endpoints to assess the efficacy of interventions. The NIA ITP is not a mechanism for funding sponsors’ laboratories to perform the work, but rather it is a collaborative effort between the three NIA-funded testing sites and the sponsors who propose interventions for study. The sponsor's role is to provide the rationale for investigating the intervention, make recommendations on the dose, route, and timing for administration of the intervention, and propose assays and measurements to document the efficacy of the intervention. The sponsors have access to all data developed from the treated mice, assist in analysis of the data, and co-author on resulting publications.

One of the important aspects of the ITP protocol is that all compounds are tested on both male and female mice. The NIH has recently begun actively promoting the recognition of sex as a biological variable in animal studies (Clayton & Collins, 2014). The ITP has seen sex differences in the response to several interventions tested, such as the recent report on three interventions, acarbose, 17-α-estradiol, and nordihydroguaiaretic acid, that have far greater effects in males than in female mice (Harrison *et al*., 2014). These findings highlight the importance of testing both sexes in intervention studies.

The NIA ITP is soliciting proposals for compounds to enter the study in 2016. The deadline for receipt of proposals is February 26, 2015. Information on the NIA ITP and guidelines for proposal development are posted at: http://www.nia.nih.gov/research/dab/interventions-testing-program-itp. Questions should be directed to Dr. Nancy Nadon (nadonn@nia.nih.gov).
